# Statements of Mentorship

**DOI:** 10.1523/ENEURO.0411-18.2018

**Published:** 2018-12-14

**Authors:** Daniel A. Colón-Ramos

**Affiliations:** 1 Department of Neuroscience, Program in Cellular Neuroscience, Neurodegeneration and Repair, Yale University School of Medicine, New Haven, CT 06510; 2 Department of Cell Biology, Yale University School of Medicine, CT 06510; 3 Instituto de Neurobiología, Recinto de Ciencias Médicas, Universidad de Puerto Rico, San Juan 00926-1117, PR

**Keywords:** Landis Award, mentor, mentoring

The Landis Award for Outstanding Mentorship was created in 2018 by the National Institute of Neurological Disorders and Stroke (NINDS) to recognize that “mentorship and training are critical to the development of exceptional future scientists” and to honor the legacy of former NINDS Director Story Landis, PhD, in mentoring generations of neuroscientists. I was honored to be nominated by my mentees and then selected by the committee as part of the inaugural group that received the award. While profoundly touched by the recognition, I am the first to admit that in mentoring, my aspirations exceed my capacities or training (and anybody who has worked in my lab will be the second to admit that). My mentorship skills are a work in progress.

Yet unlike scientific ideas, which are also “works in progress” that benefit from the critiques of our peers, mentoring approaches seldom benefit from the comments and wisdom of our colleagues and mentees. For example, I must have written, in the 10 years I have been faculty member, dozens of research statements and plans, progress reports, and grants that have all benefited from the collective wisdom of my colleagues and mentees. My science is better for it. Yet, when it comes to one of the most important activities I do, the training of mentees, it was in preparing for the Landis Award that I wrote my very first Statement of Mentorship.

To be sure, one could argue that writing a Statement of Mentorship is not necessary or even important to being a good mentor. That the tasks necessary for being a good mentor are self-evident. Some might even argue that the best way to be a good mentor is to focus solely on the scientific ideas, not so much on the individual mentee. Yet I would argue that the few times I have gotten feedback on my mentoring, I have benefited tremendously from it, and so have my mentees and the science we do together (Fig. 1). When it comes to learning, be it in mentoring or regarding new scientific ideas or techniques, I worry about the “blind spots,” that which I do not know that I do not know. The remedy for that, when it comes to scientific ideas, has been open, effective, and critical discussions with my peers. Could our mentoring, similar to our scientific ideas, benefit from the collective wisdom and experience from our colleagues and mentees?

In that spirit, below I share my lab’s Statement of Mentorship submitted to the Landis Award, not as a finished set of ideas, but as a living statement of our lab’s aspirations and to initiate a dialogue around mentorship. I also share a number of mentoring resources who Dr. Michelle Jones-London from NINDS kindly sent me.

## Statement of Mentorship

No matter who we are and no matter where we were born, we share a fundamental curiosity about the world around us. That shared human instinct does not mean we are all meant to become scientists, that we all share the same motivations, or that we think similarly about science or the world around us. But it does mean that our shared curiosity is a fundamental aspect of our humanity. This universality is foundational to my philosophy as a mentor in science. I therefore start my mentoring pledge with an aspiration to “do no harm” to my mentee’s innate interest in science.

While I recognize the universality of scientific curiosity, I also recognize that individual backgrounds shape our engagement with science. Our individual experiences differ based on the intersectionality of our identities, which includes our identity as scientists but also includes our race/ethnicity, gender identity, religion, culture, socioeconomic status, etc. These factors influence our interests, motivations, and scientific ideas. It is in the richness of this intersectionality that most of the opportunities and challenges lie for me as a mentor.

**Figure 1.**
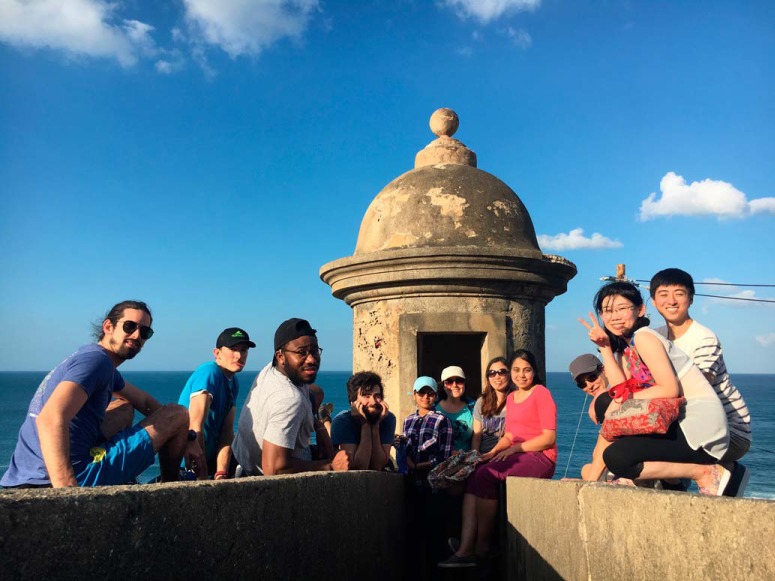
Colón-Ramos lab in the Annual Lab Retreat in San Juan, Puerto Rico (March, 2018). In 2018, after Hurricane María, we decided to conduct our annual lab retreat and workshops in San Juan, PR. From left to right: Ernesto Cabezas-Bou, Mark Moyle, Leighton Duncan, Agustin Almoril-Porras, Shavani Prashad, Sarah Hill, Sisi Yang, Titas Sengupta, Richard Ikegami, Zhao Xuan, and Joon Lee. Not in the picture: Mayra Blakey, Josh Hawk, SoRi Jang, Ian Gonzalez, Laura Manning, Milind Singh, Noelle Koonce, and Lin Shao.

Science works best to produce truly novel insights when influenced by diverse ideas from individuals working together. I intentionally create a space within my laboratory which harnesses our collective interests to build strong teams joined in a common pursuit of fundamental discoveries in science, while recognizing and celebrating our diverse backgrounds and identities. Supporting a diverse group of mentees creates both interpersonal and group dynamics that reflect issues at play in our scientific communities and, at large, concerns of racial, gender, and socioeconomic equity, for example. This means opening a lab meeting with a discussion of the “#metoo” movement in academia; it means postponing afternoon meetings after the death of Eric Garner to talk to a mentee of color about what this death represents for her, as an underrepresented scientist of color working at Yale University; it means traveling with my lab to Puerto Rico after the archipelago was devastated by Hurricane Maria to support and learn from our colleagues there (Fig. 1). In other words, recognizing, respecting, and fostering unique perspectives means challenging normative standards, not only in the scientific paradigms tackled by our research program but also in the paradigms of who and how people are meant to succeed in science. How this philosophy towards mentorship translates at the bench includes active listening to arguments, challenging assumptions, and rigorous examination of the foundations of a discovery, all while respecting the dignity of the individual and fostering their growth as a scientist. Balancing rigor while fostering growth requires investing the time needed to know and appreciate the individual perspectives of my mentees.

I have established the following mentoring approach to achieve this:

1. **Common approaches to scientific excellence by investing in the individual**. There are some fundamental skills I feel all aspiring scientists need to learn (and experienced scientists need to polish) when rigorously approaching scientific problems. Those skills include recognizing assumptions in experimental design, achieving best practices of reproducibility in quantifications and data analyses, and recognizing biases (among other skills). The way I approach mentoring and training is through structured meetings that invest heavily in the individual towards understanding their drive, their unique perspectives, and how each can build towards the development of these skills. Specifically, upon arriving at the lab, each mentee is given a binder which includes information on the lab’s mission and our mentoring philosophy. We establish weekly meetings to discuss experimental design, findings, and interpretations. These meetings are tailored to encourage independent critical thinking and project ownership, so mentees are asked to think about their experimental design, results, and interpretations prior to the meeting. Then, during the meetings, we focus on in-depth discussions in which mentees present their thoughts, identify and challenge assumptions, and conceptualize their research questions in broader contexts of overarching significance. Once a year, we have a dedicated meeting to discuss motivation and career goals, in which the mentee answers a set of questions in writing which include self-assessment of strengths and areas of improvement as a scientist, evaluation of my performance as a mentor, and their project and personal career goals. These meetings provide an opportunity to understand, not only the strengths of the mentee, but their individual needs and their intersectionality of identity in the context of their motivation and progress. The meetings provide continuous reinforcement of key precepts of the scientific process (identifying assumptions, biases and good experimental design) while also creating a mentorship framework in which the mentees progressively grow as independent thinkers and in their identity as scientists.
2. **Model by example: approaching science with rigor and humility.** In science, knowledge is important but only as a first step to recognize the boundaries of what we know and, importantly, where they end. It is therefore critical for the training scientist to feel comfortable in recognizing, with humility, their ignorance. I model this by making it clear to my mentees that I do not have all the answers, not in the research projects they are driving, and not in mentoring. Like the research projects, I approach mentoring as a team activity in which the mentor-mentee pair jointly learns how to navigate the new terrain. Being comfortable with recognizing our ignorance does not mean being complacent in mediocrity. The recognition of ignorance requires rigor and is a first step towards the recognition of opportunities in learning and growth. But in that process, while I am partial and supportive to the mentee, I am impartial and tough towards the work. I also request, and welcome, a similar treatment from the mentee towards my own ideas, biases, and assumptions.
3. **Creating a network of mentors.** Mentoring in my lab happens during individual sessions and also in sub-group meetings of teams working in conceptually related projects. The purpose of these meetings is to create a network of mutual mentoring and support. To achieve this, my lab and I have jointly brainstormed and designed activities that allow us all to grow in our roles. We recognize that being mentors or mentees, like being teachers or students, is relational, these are not fixed roles, and the roles change depending on the context. As scientists in the pursuit of knowledge, we need the mental agility to be both mentors and mentees. To learn this, all my mentees also serve as mentors to new lab members, regardless of hierarchy in training. Experience is important for being a good mentor but is not sufficient; being a good mentor also requires skills in listening and teaching. To train in mentorship, the lab and I jointly created a mentoring document in which we articulate the responsibilities of both mentors and mentees. We also have sessions to discuss how we can all be better teachers and students to each other. In that way, we move beyond the normative hierarchical structures in scientific training and instead create a web of mutual accountability and support in which responsibility and leadership is encouraged. Finally, the relationship of shared accountability between lab members is reinforced through an annual two-day lab retreat in which we discuss in depth and in an informal but stimulating setting the future of the lab (Fig. 1). In these meetings, we discuss the progression of projects, overarching conceptual questions of the field and what our lab can do to address them. The purpose of the lab retreat is to create a space away from the demands of everyday bench work to facilitate this introspection, evaluation, and discussion. Discussions in the retreat have also included career path navigation, science and society conversations on bioethics and policy, and discussions on scientific reproducibility and transparency based on the Landis et al. (2012) report.

In summary, my mentoring philosophy is based on recognizing, respecting, and investing in the individual, celebrating diversity of thought, and fostering collaborative efforts that allow us to jointly bring our unique experiences to bear in addressing the cell biology of the synapse and behavior. I work to create a lab environment which honors scientific rigor and respects the dignity of individuals to create a learning and discovery space where we are all comfortable identifying and recognizing our unique strengths and opportunities for growth.

This mentoring document was jointly prepared in collaboration with lab members who read and edited the document.

Mentoring resources:

NRMN, CienciaPR.org, or mentor training

https://www.ncbi.nlm.nih.gov/pmc/articles/PMC4477740/

Mentoring articles on SfN Neuronline

http://neuronline.sfn.org/Career-Specific-Topics/Professional-Development

How to Get the Mentoring You Want: A Guide for Graduate Students at a Diverse University

http://www.rackham.umich.edu/downloads/publications/mentoring.pdf

Making the Right Moves and Training Scientists to Make the Right Moves

http://www.hhmi.org/programs/resources-early-career-scientist-development

Individual Development Plan (IDP), a Web-based career-planning tool created to help graduate students and postdocs in the sciences define and pursue their career goals

http://myidp.sciencecareers.org/

National Research Mentoring Network

https://nrmnet.net/

Mentoring Compacts:

Example compacts for download are available at

https://ictr.wisc.edu/mentoring/mentoring-compactscontracts-examples/

## Synthesis

Reviewing Editor: Esther Stoeckli, University of Zurich Decisions are customarily a result of the Reviewing Editor and the peer reviewers coming together and discussing their recommendations until a consensus is reached. When revisions are invited, a fact-based synthesis statement explaining their decision and outlining what is needed to prepare a revision will be listed below.

Thank you for the manuscript summarizing your mentoring philosophy and congratulations for being one of the winners of the Landis Mentoring Award. I like your thoughts about 'mentoring as work in progress' and mentoring as a two-way street that involves the mentor but also the mentee. I think your commentary will be inspiration for the readers or, hopefully, confirmation that their own mentoring strategies are going in the right direction. Also, I like the links to further information about good mentoring practices added to your article.
